# The Utilization of Patient-Reported Outcome Measures in Assessing the Treatment of Osteochondral Lesions of the Ankle Versus the Knee

**DOI:** 10.1177/03635465251333088

**Published:** 2025-06-07

**Authors:** Darius Luke Lameire, Caroline Cristofaro, Jong Min Lee, Kathrine Bhargava, Shgufta Docter, David Wasserstein, Sam Si-Hyeong Park

**Affiliations:** * Division of Orthopaedic Surgery, Department of Surgery, University of Toronto, Toronto, Ontario, Canada; † Division of Orthopaedic Surgery, William Osler Health System, Etobicoke, Ontario, Canada; ‡ Department of Laboratory Medicine and Pathobiology, University of Toronto, Toronto, Ontario, Canada; § University of Limerick School of Medicine, Limerick, Ireland; ‖ Division of Orthopaedic Surgery, Department of Surgery, Women’s College Hospital, Toronto, Ontario, Canada; ¶ Division of Orthopaedic Surgery, Department of Surgery, Sunnybrook Health Sciences Centre, Toronto, Ontario, Canada; # University of Toronto Orthopaedic Sports Medicine Program, Toronto, Ontario, Canada; ** University of Toronto Orthopaedic Foot and Ankle Program, Toronto, Ontario, Canada; Investigation performed at the Division of Orthopaedic Surgery, Department of Surgery, University of Toronto, Toronto, Ontario, Canada

**Keywords:** ankle, knee, osteochondral, outcomes, valid, PROMs

## Abstract

**Background:**

When assessing the outcomes of ankle and knee osteochondral lesions (OCLs), there are numerous patient-reported outcome measures (PROMs) that are used; however, not all are validated.

**Purpose:**

To compare the utilization of PROMs in assessing the treatment of ankle OCLs versus knee OCLs.

**Study Design:**

Systematic review; Level of evidence, 4.

**Methods:**

A systematic search of Embase, MEDLINE, and CINAHL was conducted to identify all observational or experimental studies from January 1, 2014 to December 31, 2023 that used PROMs to assess the treatment of ankle or knee OCLs. The frequency of the use of specific validated PROMs between the ankle OCL and knee OCL literature was compared using an independent *t*-test. Correlation coefficients were calculated to assess differences based on journal impact factor (divided into quartiles), publication year, or level of evidence.

**Results:**

A total of 233 eligible ankle OCL studies and 211 knee OCL studies were identified. Validated clinical outcome measures were used in 41.2% of ankle OCL studies compared with 87.7% of knee OCL studies (*P* < .001). There were a total of 44 outcome measures used in ankle OCL studies, with the majority of studies (67.8%) utilizing the AOFAS (American Orthopaedic Foot and Ankle Society) score. There were no correlations between the use of validated outcome measures in the ankle OCL studies and journal impact factor (*P* = .78), publication year (*P* = .16), or level of evidence (*P* = .45). Similarly, there were no correlations for the knee OCL studies based on journal impact factor (*P* = .60), publication year (*P* = .25), or level of evidence (*P* = .55).

**Conclusion:**

Validated clinical outcome measures were more frequently utilized in knee OCL studies compared with ankle OCL studies. The low frequency of validated outcome measures used within the ankle literature may limit how well treatment effectiveness in the management of ankle OCLs is evaluated.

Functional outcome reporting in foot and ankle surgery has evolved significantly over the past 20 years.^33^ Historically, outcome reporting relied on clinician-reported outcome measures based on objective findings such as range of motion, deformity, swelling, strength, and radiographic appearance. Newer patient-reported outcome measures (PROMs) rely on patient self-reporting to obtain a direct reflection of patients’ perception of their functionality, health, and quality of life after treatment.^33^ While both clinician-reported outcome measures and PROMs can provide useful information, it is thought that PROMs provide more accurate details on patients’ function in the long term.^18,33^

Currently, there are >80 PROMs available for foot and ankle abnormalities.^24^ However, very few of these outcome measures have been validated. There is no clear consensus on which outcome measure is the most optimal to use for specific abnormalities including osteochondral lesions (OCLs) of the ankle. As a result, many clinicians have continued to utilize the American Orthopaedic Foot and Ankle Society (AOFAS) score for the past 15 years, despite it being a nonvalidated clinician-reported outcome measure with poor reliability for patients with general ankle abnormalities.^18,32^ In stark contrast, for the knee, there are multiple scoring instruments that are validated and commonly used, including the Knee injury and Osteoarthritis Outcome Score (KOOS), International Knee Documentation Committee (IKDC) subjective knee form, Patient-Reported Outcomes Measurement Information System (PROMIS), and Lysholm knee scoring scale, to assess patients’ functional outcomes for general knee conditions and/or specifically OCLs.^8,13,14,21,23,31,34^

In the foot and ankle, there are a few validated measures to assess objective and subjective functional outcomes for OCLs including the Foot and Ankle Outcome Score (FAOS), Foot and Ankle Ability Measure (FAAM), and Foot Function Index (FFI).^7,25,32,33,35,38^ The FAOS has shown evidence of validity, reliability, and responsiveness in evaluating outcomes in patients with ankle OCLs.^7,38^ The FAAM has been shown to be a valid and reliable measure of assessing physical function for general lower leg, foot, and ankle disorders.^25^ Additionally, the FFI is a valid measure of health status outcomes in patients with foot and ankle disorders.^35^

The primary purpose of this systematic review was to compare the utilization of PROMs in assessing the treatment of ankle OCLs versus knee OCLs. The ankle and knee joints were selected because they are the most frequently encountered locations for OCLs in the lower extremity.^12^ The secondary objective was to determine if there was a correlation between the use of validated outcome measures and journal impact factor, publication year, or level of evidence.

## Methods

**Figure 1.**
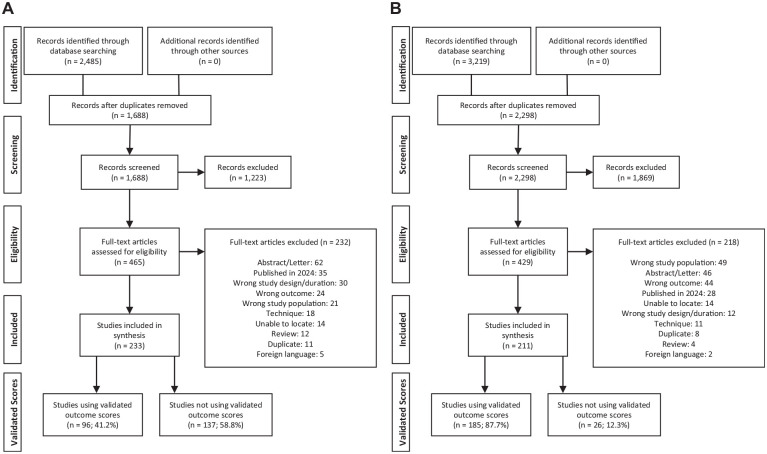
PRISMA diagrams. (A) Ankle osteochondral lesion (OCL) studies. (B) Knee OCL studies.

We conducted a systematic review in accordance with the PRISMA (Preferred Reporting Items for Systematic Reviews and Meta-Analyses) guidelines (Figure 1).^26^ This study was not registered in PROSPERO.

### Search Strategy

Two investigators (D.L.L. and C.C.) independently performed a systematic search of the Embase, MEDLINE, and CINAHL electronic databases. There were two keyword searches performed, with one search of the ankle literature and the other search of the knee literature (see Appendices 1 and 2, available in the online version of this article). The search terms for the ankle literature included the following: “osteochondral,” “osteochondral lesions,” “osteochondral defect,” “osteochondritis dissecans,” “cartilage injury,” “cartilage lesions,” “ankle,” “ankle joint,” and “talus.” The search terms for the knee literature included a combination of the following: “osteochondral,” “osteochondral lesions,” “osteochondral defect,” “osteochondritis dissecans,” “knee,” “knee joint,” and “patellofemoral joint.” The systematic search was performed on August 8, 2024 for studies published between January 1, 2014 and December 31, 2023.

### Eligibility Criteria

Studies were eligible for inclusion if they met the following criteria: (1) level I to IV studies that evaluated functional outcomes in patients undergoing nonoperative or operative treatment for OCLs or osteochondritis dissecans, (2) the primary location of the abnormality was at the knee joint or ankle joint, and (3) studies that were published between January 1, 2014 and December 31, 2023. Studies were excluded if there were <10 patients being treated for OCLs or osteochondritis dissecans.

### Study Selection and Data Extraction

Two independent reviewers (D.L.L. and C.C.) screened the titles and abstracts from the initial literature search and extracted relevant data from all eligible studies into a standardized collection sheet using Google Sheets (Alphabet Inc, USA). Data collected included the functional outcome measures used, journal, publication year, study design, and level of evidence. A separate search was also performed through Journal Citation Reports to identify and record the impact factors of the journals.^1^ The most recent journal impact factors available on the Clarivate website (www.clarivate.com) in September 2024 were utilized. The scores of all journals were compared and divided into quartiles based on the impact factor (see Appendices 3 and 4, available online). Any ties were decided based on journal impact factors without self-citations.

### Validated Outcome Measures

Validated outcome measures for ankle OCLs included the FAOS, FAAM, and FFI.^7,25,32,33,35,38^ For knee OCLs, validated outcome measures included the Lysholm scale, IKDC form, KOOS, and PROMIS.^8,13,14,21,23,31,34^

### Statistical Analysis

The frequency and proportion of validated functional outcome measures used in each study were calculated and compared between the ankle and knee literature using an independent *t*-test. The Pearson correlation coefficient was used to determine whether there was an association between journal impact factor (divided into quartiles) or publication year and the frequency of the use of validated measures. The Spearman rank correlation coefficient was used to assess if there was an association with level of evidence. Statistical significance was defined as *P* < .05. SPSS software (Version 25; IBM) was used for all statistical analyses.

## Results

The results of our initial search generated 2,485 ankle OCL studies and 3,219 knee OCL studies for review. After title and abstract screening, 465 ankle OCL studies and 429 knee OCL studies remained. After a full-text review for study eligibility, there were a total of 233 ankle OCL studies (Figure 1A) and 211 knee OCL studies (Figure 1B). The ankle OCL literature consisted of seven randomized controlled trials, 10 case-control studies, 69 cohort studies, and 147 case series. The knee OCL literature consisted of nine randomized controlled trials, eight case-control studies, 65 cohort studies, and 129 case series.

### Functional Outcome Measures

A total of 44 different functional outcome measures were reported in the 233 ankle OCL studies. The 20 most commonly utilized measures are listed in Table 1. The most frequently used were the AOFAS score (67.8% of studies), visual analog scale (VAS) for pain (64.8% of studies), and FAOS (27.0% of studies). The FAAM was used in 10.3% of studies, and the FFI was used in 6.9% of studies. Overall, 96 ankle OCL studies (41.2%) utilized at least one validated outcome measure.

**Table 1:** Top 20 Most Commonly Reported Functional Outcome Measures for Ankle OCL Studies*^a^*

Rank	Measure	No. of Times Reported (% of Studies)
1	AOFAS Ankle-Hindfoot Score	158 (67.8)
2	Visual Analog Scale (VAS) for Pain	151 (64.8)
3	Foot and Ankle Outcome Score*^b^*	63 (27.0)
4	Tegner Activity Scale	27 (11.6)
5	Foot and Ankle Ability Measure*^b^*	24 (10.3)
T6	Short Form–12	21 (9.0)
T6	Short Form–36	21 (9.0)
8	Foot Function Index*^b^*	16 (6.9)
9	Ankle Activity Score	9 (3.9)
10	EQ-5D	5 (2.1)
T11	Karlsson Ankle Function Scale	4 (1.7)
T11	PROMIS	4 (1.7)
T13	Hannover Scoring System	3 (1.3)
T13	Japanese Society for Surgery of the Foot	3 (1.3)
T13	Karlsson-Peterson scale	3 (1.3)
T13	Manchester-Oxford Foot Questionnaire	3 (1.3)
T17	Ankle Osteoarthritis Scale	2 (0.9)
T17	Berndt and Harty scale	2 (0.9)
T17	European Foot and Ankle Society score	2 (0.9)
T17	5 other functional outcome measures	2 (0.9)

a AOFAS, American Orthopaedic Foot and Ankle Society; OCL, osteochondral lesion; PROMIS, Patient-Reported Outcomes Measurement Information System.

b Validated outcome measures.

Within the 211 knee OCL studies, 29 different outcome measures were reported. The 20 most commonly reported outcome measures can be found in Table 2. The most frequently used were the IKDC form (64.5% of studies), KOOS (37.4% of studies), and Tegner Activity scale (31.3% of studies). The Lysholm scale was used in 24.6% of studies, and PROMIS was used in 2.8% of studies. Overall, 185 knee OCL studies (87.7%) utilized at least one validated outcome measure, which represented a significant statistical difference from the ankle OCL literature (*P* < .001).

**Table 2:** Top 20 Most Commonly Reported Functional Outcome Measures for Knee OCL Studies*^a^*

Rank	Measure	No. of Times Reported (% of Studies)
1	IKDC Subjective Knee Form*^b^*	136 (64.5)
2	KOOS*^b^*	79 (37.4)
3	Tegner Activity Scale	66 (31.3)
4	Visual Analog Scale (VAS) for Pain	65 (30.8)
5	Lysholm Knee Scoring Scale*^b^*	52 (24.6)
T6	Short Form–36	21 (10.0)
T6	WOMAC	21 (10.0)
8	Short Form–12	19 (9.0)
9	Knee Outcome Survey – Activities of Daily Living Scale	15 (7.1)
10	Marx Activity Rating Scale	14 (6.6)
11	Cincinnati Sports Activity Scale	13 (6.2)
12	Kujala Score	12 (5.7)
13	Knee Society Function Score	10 (4.7)
14	Merle d’Aubigné-Postel Score	9 (4.3)
15	Single Assessment Numeric Evaluation	7 (3.3)
T16	PROMIS*^b^*	6 (2.8)
T16	Hannover Scoring System	6 (2.8)
T18	Tegner Lysholm Knee Score	5 (2.4)
T18	Veterans RAND 12-Item Health Survey	5 (2.4)
20	2 other functional outcome measures	3 (1.4)

a IKDC, International Knee Documentation Committee; KOOS, Knee injury and Osteoarthritis Outcome Score; OCL, osteochondral lesion; PROMIS, Patient-Reported Outcomes Measurement Information System; WOMAC, Western Ontario and McMaster Universities Osteoarthritis Index.

b Validated outcome measures.

### Journal Impact Factor

The ankle OCL studies were published across 48 different journals. The journals with the greatest number of ankle OCL publications were *Foot & Ankle International* (15.9%), *Knee Surgery, Sports Traumatology, Arthroscopy* (13.7%), and *The American Journal of Sports Medicine* (9.4%). There was no correlation between the use of validated outcome measures and the journal impact factor (*P* = .78).

The knee OCL studies were published across 39 different journals. The journals with the greatest number of knee OCL publications were *The American Journal of Sports Medicine* (21.3%), *Cartilage* (10.9%), and *Knee Surgery, Sports Traumatology, Arthroscopy* (10.4%). There was no correlation between the use of validated outcome measures and the journal impact factor (*P* = .60).

### Publication Year

**Figure 2.**
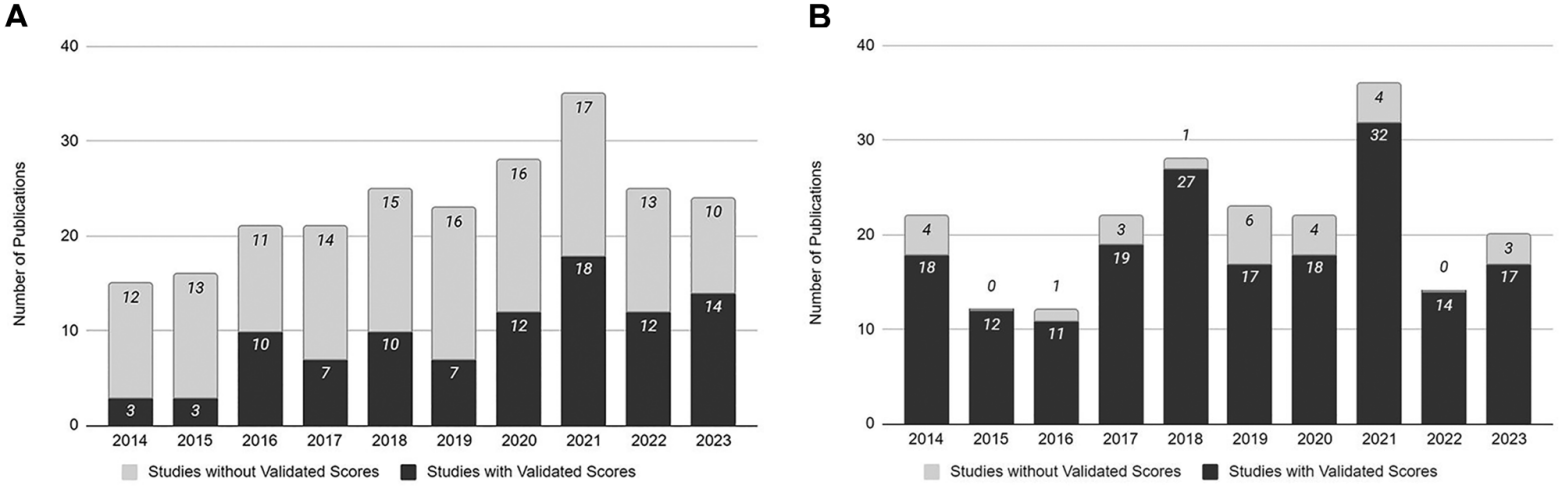
Usage of validated functional outcome measures based on publication year. (A) Ankle osteochondral lesion (OCL) literature. (B) Knee OCL literature.

The publication rates in both the ankle OCL and knee OCL literature were consistent throughout the study period of 2014 and 2023 (Figure 2). There was no correlation between the use of validated outcome measures and the publication year in the ankle OCL literature (*P* = .16) (Figure 2A) or knee OCL literature (*P* = .25) (Figure 2B).

### Levels of Evidence

**Figure 3.**
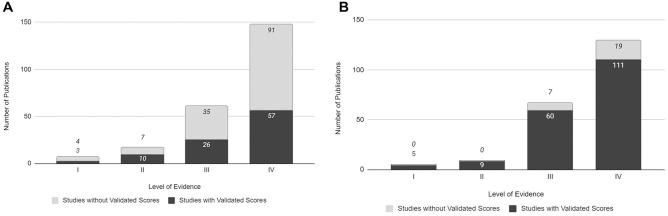
Usage of validated functional outcome measures based on level of evidence. (A) Ankle osteochondral lesion (OCL) literature. (B) Knee OCL literature.

Case series was the most common study design in both ankle OCL (Figure 3A) and knee OCL (Figure 3B) studies. There was no correlation between the use of validated outcome measures and the level of evidence in the ankle OCL studies (*P* = .45) or knee OCL studies (*P* = .55).

## Discussion

This study identified significant heterogeneity with regard to the functional outcome measures used in the ankle OCL literature, with 44 different measures reported. Only 41.2% of ankle OCL studies from 2014 to 2023 utilized validated outcome scoring systems. This was significantly different from the knee OCL literature in which 87.7% of the studies from 2014 to 2023 utilized at least one validated outcome measure. To our knowledge, this study is the first to compare the utilization of validated PROMs in assessing the management of OCLs of the ankle and the knee. The underutilization of validated PROMs in the ankle literature raises significant concern about current available “evidence-based” outcomes and treatment algorithms for the management of OCLs of the ankle.

A significant issue in the ankle OCL literature was the high dependence on the AOFAS score (reported in 67.8% of studies) in evaluating clinical outcomes. However, this is consistent with much of the general foot and ankle literature.^24,32^ The rationale provided by many authors in using the AOFAS score is that it has been used in the majority of the literature and it allows for a direct comparison of outcomes.^29^ However, as highlighted by the position statement from the AOFAS in 2011, the AOFAS score is not a validated and reliable outcome scoring system.^28^ It is largely clinician driven and not specific to different abnormalities of the foot and ankle, including OCLs.^18,28,33^ Despite this, the AOFAS score continues to be utilized. The frequent use of nonvalidated PROMs is also found in other orthopaedic subspecialty literature, such as in pediatrics.^6,27,37^ Yet, we found that the large majority (87.7%) of studies assessing the management of knee OCLs used at least one validated PROM, with the two most commonly used being the IKDC form (64.5% of studies) and the KOOS (37.4% of studies). This increased uniformity in reporting outcomes based on validated measures helps to improve the quality and reliability of the studies to support evidence-based conclusions regarding treatment methods.

Our findings also showed that the underutilization of validated outcome measures in the ankle OCL literature was widespread across many factors. No statistical correlation was seen between the use of validated measures and the journal impact factor or publication year. This status quo may suggest that the lack of validated outcome measures in ankle OCL studies is not perceived as an issue, is not recognized, or is simply generally accepted despite the study quality. However, stronger evidence-based studies will help to better guide the treatment and outcomes of patients. This may be a pervasive issue in the foot and ankle literature because, to our knowledge, there have been no assessments on the use of validated outcome measures in the literature evaluating other foot and ankle procedures.

The knee OCL literature showed that validated PROMs could help to reduce heterogeneity and provide better evidence-based treatment options. Additionally, there was high usage of validated outcome measures, regardless of the journal impact factor or publication year. Interestingly, there was no statistical difference between the use of validated outcome measures based on the level of evidence of studies assessing either knee OCLs or ankle OCLs. In the knee OCL literature, 100.0% (14/14) of level 1 and 2 studies used validated outcome measures, whereas only 54.2% (13/24) of level 1 and 2 studies assessing ankle OCLs used validated outcome measures.

The use of validated PROMs has intuitive value when assessing patient outcomes in primary studies, but it is important to evaluate the broader clinical impact that it may have. Validated PROMs in the knee OCL literature has likely contributed to recommendations from esteemed orthopaedic organizations and knee societies. The American Academy of Orthopaedic Surgeons (AAOS) released a 2023 update of its 2010 clinical practice guideline on the treatment and diagnosis of osteochondritis dissecans.^3^ In the updated guideline, the AAOS reassessed previous guidelines and upgraded its recommendation for the option of surgery for skeletally mature patients with salvageable unstable osteochondritis dissecans lesions from a consensus to limited agreement.^3^ The initial recommendation was based on a limited number of studies available at that time. In the update, the AAOS cited additional supporting evidence, including from studies published in 2020^10^ and 2023^19^ that found improvements in validated outcome measures for the treatment of knee OCLs. As such, the use of validated PROMs can impact clinical practice through stronger recommendations and guidelines. Additionally, as there are still studies published without the use of validated measures, and as there was no statistical difference in the use of validated PROMs based on the journal impact factor, the use of these validated measures may be encouraged by journals, which will aid to further strengthen findings.

Regarding guidelines for ankle OCLs, in 2022, the AOFAS released a position statement on the use of osteochondral transplantation for the treatment of OCLs of the talus.^5^ Based on the outcomes from multiple studies, the AOFAS endorsed the use of autograft and allograft transplantation, particularly for patients with large diameter lesions, those with cystic lesions, and those who have failed previous surgical treatment.^5^ Specifically, it reports multiple studies^20,22,30^ that found improvements in AOFAS ankle-hindfoot scores and VAS for pain scores; however, it only cites one study^2^ that found significant improvements in FAAM scores. As such, by utilizing more validated outcome measures in primary research studies, it may further improve the strength and robustness of recommendations for the treatment of ankle OCLs, as has been seen in the knee OCL literature.

One potential alternative to disease-specific PROMs is PROMIS, a generic outcome assessment instrument administered electronically as a computerized adaptive test.^16^ PROMIS has already been validated for patients with knee articular cartilage defects.^31^ Furthermore, PROMIS Physical Function was first validated for common foot and ankle conditions in 2013.^16^ Preoperative PROMIS Physical Function and Pain Interference scores have been shown to predict postoperative improvements in corresponding domains, and PROMIS Physical Function was subsequently validated to predict postoperative improvement in patients undergoing foot and ankle surgery.^4,15^ In addition, both PROMIS Physical Function and Pain Interference scores were found to be sensitive and responsive to changes in patients’ health in the study by Hung et al.^17^ Based on the current evidence, PROMIS Physical Function and Pain Interference are excellent tools to consider for patients with ankle OCLs and warrant significant consideration as consensus choices for functional outcome reporting of ankle OCLs.

The key strengths of this systematic review include the comprehensive and rigorous search and article screening process. Another strength of this review is that it highlights the underutilization of validated outcome measures in the ankle OCL literature, especially when compared with the knee OCL literature. The limitations of the review are reflective of the few validated measures available for assessing ankle OCLs, with only three validated outcome measures. Our review also did not look specifically at whether the treatment of OCLs resulted in improved or worse outcomes for patients within each study, as the primary objective of this review was to determine how frequently validated outcome measures were used. Finally, there was significant heterogeneity in the terminology of OCLs of the ankle. We elected to include osteochondritis dissecans, focal cartilage defect, and osteochondral fracture under the same umbrella term of “osteochondral lesion” in our study. These inclusions may not be agreed upon universally within the orthopaedic community.

The use of validated outcome measures to assess the management of various orthopaedic conditions is important. This may be even more critical in the management of ankle OCLs. Many of the current treatment algorithms and surgical options for ankle OCLs follow similar principles as for knee OCLs. However, it is well known that ankle cartilage has different biomechanical and biochemical properties than the knee with its unique multiaxial joint orientation.^9,36^ Thus, we must ensure that the effectiveness of current treatment approaches for ankle OCLs are assessed accurately. A survey of surgeons by the AOFAS found that “cartilage reconstruction” was the number one area of research that needed investigative support.^11^ This may reflect inconsistent or poor outcomes with current treatment options for ankle OCLs or may reflect the evidence within the literature. Pinski et al^29^ highlighted that there are currently low levels of evidence and low methodological quality of clinical outcome studies on cartilage repair of the ankle. The underutilization of validated outcome measures further compromises how we assess the effectiveness of current treatment methods for ankle OCLs.

## Conclusion

Within ankle OCL studies, validated outcome measures were significantly less utilized compared with knee OCL studies. Approximately two-thirds of the ankle OCL studies utilized the AOFAS score, despite the measure not being shown to be valid or reliable in this patient population. The low frequency of validated measures used within the ankle literature may limit how well treatment effectiveness in ankle OCLs is appropriately evaluated. Improved uniform reporting using validated outcome measures within the ankle OCL literature will help to achieve better evidence-based conclusions and treatment algorithms to enhance patient care.

## Supplemental Material

sj-pdf-1-ajs-10.1177_03635465251333088 – Supplemental material for The Utilization of Patient-Reported Outcome Measures in Assessing the Treatment of Osteochondral Lesions of the Ankle Versus the Knee Supplemental material, sj-pdf-1-ajs-10.1177_03635465251333088 for The Utilization of Patient-Reported Outcome Measures in Assessing the Treatment of Osteochondral Lesions of the Ankle Versus the Knee by Darius Luke Lameire, Caroline Cristofaro, Jong Min Lee, Kathrine Bhargava, Shgufta Docter, David Wasserstein and Sam Si-Hyeong Park in The American Journal of Sports Medicine
