# Supplementary material for: The Utilization of Patient-Reported Outcome Measures in Assessing the Treatment of Osteochondral Lesions of the Ankle Versus the Knee
Source: Am J Sports Med. 2025 Jun 7;53(14):3565–71. doi: 10.1177/03635465251333088 (PMC12657660; doi:10.1177/03635465251333088)
Supplement: sj-pdf-1-ajs-10.1177_03635465251333088 – Supplemental material for The Utilization of Patient-Reported Outcome Measures in Assessing the Treatment of Osteochondral Lesions of the Ankle Versus the Knee [file sj-pdf-1-ajs-10.1177_03635465251333088.pdf]

Utilization of Patient-Reported Outcome Measures in Assessing Treatment of Osteochondral Lesions of the Ankle versus the Knee

Appendix 1: Ankle OCL Search Strategy

Medline	Embase	CINAHL
1 osteochond*.mp. (28266)<br>2 lesion*.mp. (1016011)<br>3 defect*.mp. (665260)<br>4 2 or 3 (1648012)<br>5 1 and 4 (9676)<br>6 exp Osteochondritis/ or exp Osteochondritis Dissecans/ or osteochondritis.mp. (8173)<br>7 5 or 6 (15711)<br>8 ankle.mp. or exp Ankle Joint/ or exp Ankle/ (85335)<br>9 talar*.mp. (5795)<br>10 exp Talus/ or talus*.mp. (8074)<br>11 8 or 9 or 10 (90966)<br>12 distal.mp. (276297)<br>13 tibia*.mp. (130860)<br>14 12 and 13 (16150)<br>15 11 or 14 (103359)<br>16 7 and 15 (2256)<br>17 limit 16 to (english language and humans and yr="2014 - 2024") (839)	1 osteochond*.mp. (30271)<br>2 lesion*.mp. (1601740)<br>3 defect*.mp. (1159528)<br>4 2 or 3 (2648394)<br>5 1 and 4 (12146)<br>6 exp Osteochondritis/ or exp Osteochondritis Dissecans/ or osteochondritis.mp. (9068)<br>7 5 or 6 (18385)<br>8 ankle.mp. or exp Ankle Joint/ or exp Ankle/ (134742)<br>9 talar*.mp. (7857)<br>10 exp Talus/ or talus*.mp. (10973)<br>11 8 or 9 or 10 (142227)<br>12 distal.mp. (406024)<br>13 tibia*.mp. (171300)<br>14 12 and 13 (24312)<br>15 11 or 14 (161007)<br>16 7 and 15 (2960)<br>17 limit 16 to (english language and humans and yr="2014 - 2024") (1480)	1. (MH "Osteochondiritis") OR (MH "Osteochondritis Dissecans") OR "Osteochondral OR osteochondritis (1,447)<br>2. (MH "Talus") OR (MH "Ankle") OR (MH "Ankle Joint") OR "osteochondral OR osteochondritis" (14,665)<br>3. 1 AND 2 (280)<br>4. Limit 3 to publication date: 20140101-20241231 (166)

Appendix 2: Knee OCL Search Strategy

Medline	Embase	CINAHL
1 osteochond*.mp. (28270)<br>2 lesion*.mp. (1016131)<br>3 defect*.mp. (665355)<br>4 2 or 3 (1648223)<br>5 1 and 4 (9677)<br>6 exp Osteochondritis/ or exp Osteochondritis Dissecans/ or osteochondritis.mp. (8174)<br>7 5 or 6 (15712)<br>8 knee.mp. or exp Knee/ or exp Knee Joint/ (216238)<br>9 exp Patellofemoral Joint/ or patellofemoral.mp. (8983)<br>10 patella.mp. or exp patella/ (18116)<br>11 8 or 9 or 10 (223046)<br>12 7 and 11 (3990)<br>13 limit 12 to (english language and humans and yr="2014 - 2024") (967)	1 osteochond*.mp. (30271)<br>2 lesion*.mp. (1601740)<br>3 defect*.mp. (1159528)<br>4 2 or 3 (2648394)<br>5 1 and 4 (12146)<br>6 exp Osteochondritis/ or exp Osteochondritis Dissecans/ or osteochondritis.mp. (9068)<br>7 5 or 6 (18385)<br>8 knee.mp. or exp Knee/ or exp Knee Joint/ (319470)<br>9 exp Patellofemoral Joint/ or patellofemoral.mp. (12871)<br>10 patella.mp. or exp patella/ (29105)<br>11 8 or 9 or 10 (329837)<br>12 7 and 11 (5509)<br>13 limit 12 to (human and english language and yr="2014 - 2024") (2076)	1. (MH "Osteochondiritis") OR (MH "Osteochondritis Dissecans") OR "Osteochondral OR osteochondritis (1,447)<br>2. (MH "Knee Joint") OR (MH "Patella") OR (MH "Knee") OR *knee OR patella OR patellofemoral) (32,705)<br>3. 1 AND 2 (383)<br>4. Limit 3 to publication date: 20140101-20241231 (176)

Appendix 3: Ankle OCL Journal Impact Quartiles

Journal	Most Recent Reported Impact Factor
Quartile 1:	
Chinese medical journal	7.5
Cells	5.1
The bone & joint journal	4.9
European radiology	4.7
Arthroscopy: the journal of arthroscopic & related surgery	4.4
The Journal of bone and joint surgery	4.4
The American Journal of Sports Medicine	4.2
Scientific reports	3.8
Annals of Translational Medicine	3.6
Knee surgery, sports traumatology, arthroscopy	3.3
Journal of Clinical Medicine	3
Quantitative Imaging in Medicine and Surgery	2.9
Quartile 2:	
Journal of orthopaedic surgery and research	2.8
Cartilage	2.7
Journal of ISAKOS	2.7
BioMed research international	2.6
Foot & ankle international	2.4
Orthopaedic Journal of Sports Medicine	2.4
Orthopaedics & traumatology, surgery & research	2.3
BMC musculoskeletal disorders	2.2
Medical science monitor	2.2
Clinical journal of sport medicine	2.1
Swiss Medical Weekly	2.1

Current medical science	2
Quartile 3:	
Archives of orthopaedic and trauma surgery	2
International orthopaedics	2
Joint diseases and related surgery	1.9
Foot and ankle surgery	1.9
Joint diseases and related surgery	1.9
Chinese journal of traumatology	1.8
Foot & ankle specialist	1.8
Orthopaedic surgery	1.8
Journal of orthopaedic science	1.5
European journal of orthopaedic surgery & traumatology	1.4
Journal of orthopaedic surgery (Hong Kong)	1.3
Medicine	1.3
Quartile 4:	
The Journal of foot and ankle surgery	1.3
Journal of sports medicine and physical fitness	1.2
Acta orthopaedica et traumatologica turcica	1.1
Operative Orthopadie und Traumatologie	1
Journal of the American Podiatric Medical Association	0.5
Konuralp Tip Dergisi	0.3
Current Orthopaedic Practice	0.2
International Journal of Clinical and Experimental Medicine	0.2
Eastern Journal of Medicine	NA
Foot	NA
Journal of Cartilage and Joint Preservation	NA
Wiadomosci lekarskie	NA

NA: Not available; ISAKOS: International Society of Arthroscopy, Knee Surgery and Orthopaedic Sports Medicine.

Appendix 4: Knee OCL Journal Impact Quartiles

Journal	Most Recent Reported Impact Factor
Quartile 1:	
The Bone and Joint Journal	4.9
Drug Design, Development and Therapy	4.7
Arthroscopy: The Journal of Arthroscopic and Related Surgery	4.4
The Journal of Bone and Joint Surgery	4.4
Tissue Engineering and Regenerative Medicine	4.4
Journal of Materials Science: Materials in Medicine	4.2
The American Journal of Sports Medicine	4.2
Biomedical Materials	3.9
Knee Surgery, Sports Traumatology, Arthoscopy	3.3
Journal of Orthopaedics and Traumatology	3
Quartile 2:	
Journal of Clinical Medicine	3
Journal of Orthopaedic Surgery and Research	2.8
The Journal of the American Academy of Orthopaedic Surgeons	2.8
Cartilage	2.7
BioMed Research International	2.6
Orthopaedic Journal of Sports Medicine	2.4
The Orthopaedic Journal of Sports Medicine	2.4
BMC Musculoskeletal Disorders	2.3
Orthopaedics and Traumatology: Surgery and Research	2.3
Injury	2.2
Quartile 3:	
Arthroscopy, Sports Medicine, and Rehabilitation	2.1
Journal of Orthopaedic Research	2.1

Archives of Orthopaedic and Trauma Surgery	2
International Orthopaedics	2
Joint Diseases and Related Surgery	1.9
Journal of Experimental Orthopaedics	1.8
Musculoskeletal Surgery	1.8
Journal of Knee Surgery	1.6
The Journal of Knee Surgery	1.6
The Knee	1.6
Quartile 4:	
Journal of Orthopaedics	1.5
European Journal of Orthopaedic Surgery and Traumatology	1.4
Journal of Orthopaedic Surgery	1.3
Acta orthopaedica et traumatologica turcica	1.1
Journal of Cartilage and Joint Preservation	0.9
Journal of Biological Regulators and Homeostatic Agents	0.8
Acta Orthopaedica Belgica	0.5
Journal of IMAB	0.1
Journal of Clinical Orthopaedics and Trauma	NA

NA: Not available; IMAB: International Medical Association Bulgaria
